# Clinical Impact of Nasal Obstructive Syndrome and Its Current Management Strategies

**DOI:** 10.7759/cureus.90762

**Published:** 2025-08-22

**Authors:** Freddy Lizano Guevara, Alberto Rojas Peláez, Edgardo J Soto-Junco, David Sáenz Araya, Enmanuel Sevilla Torres, Santiago Daniel Baizan Orias

**Affiliations:** 1 General Medicine, Universidad de Ciencias Médicas (UCIMED), San José, CRI; 2 General Medicine, Universidad de Costa Rica, San José, CRI

**Keywords:** allergic, nasal obstruction, nasal obstruction syndrome, obstructive sleep apnea, paranasal sinuses, quality of life, rhinitis

## Abstract

Nasal obstruction syndrome (NOS) is inherently complex due to the combination of nasal anatomy and physiology and pathophysiologic processes that together affect airflow, filtering, smell, and the general health of the respiratory system. The nasopharynx consists of different structures together: the septum, turbinates, and nasal valves that together perform the jobs associated with the nasal polyp, which is to regulate air conditioning and mucociliary clearance. Changes that can be recognized in the septum, turbinates, and nasal valve will individually and/or collectively affect the potential for airway obstruction. However, obstructions may not occur as a function of anatomy; they may occur via anatomical functional restrictions, as is believed to be the case with breathing-facilitating trigeminal nerve dysfunction, evidenced by the subjective sensations of nasal obstruction while no anterior nasal obstruction is observed. In addition to anatomical changes such as turbinate hypertrophy, outpt septal deviation, and/or nasal valve collapse, there are also chronic inflammatory disease states such as rhinosinusitis, allergic and non-allergic rhinitis, and nasal polyposis that will develop to produce nasal obstructions via mucosal edema and structural room through tissue remodeling.

The clinical consequence of NOS is nasal airway congestion, hyposmia, and compensatory mouth breathing, with the latter two activities causing harm to sensory deficits such as taste, sleep quality, and cognitive functioning, and impedance in health-related quality of life. Commonly utilized diagnostic procedures include nasal endoscopy, CT, and testing for the effects of nasal obstruction (e.g., the Nasal Obstruction Symptom Evaluation (NOSE) scale, Sino-Nasal Outcome Test-22 (SNOT-22), and Visual Analog Scale (VAS)), which are specific to confirm the effect of nasal obstruction. Radiological and nasal endoscopic findings focused on anatomical distortions and specific patterns of obstructive nasal difficulty, particularly in chronic, difficult-to-treat rhinosinusitis and nasal valve obstruction.

Management plans unite both pharmacologic options, such as antihistamines, corticosteroids, and immunotherapies, with surgical procedures, which can include septoplasty, turbinate reduction, nasal valve reconstruction, and functional endoscopic sinus surgery (FESS). Treatment will depend on the patient's specific medical and social history, which is especially critical for children, older-age patients, and patients with comorbid respiratory problems such as asthma or obstructive sleep apnea (OSA). For children, the typical catalyst for nasal obstruction appears to be adenoid hypertrophy, whereas older patients may differ in their nasal microbiota. Management is a multidisciplinary team effort, engaging otolaryngology, allergy, and pulmonology specialists to treat this multifaceted condition.

## Introduction and background

The clinical relevance of nasal obstruction syndrome (NOS) in healthcare practice is meaningful, as it has many physiological, functional, and systemic ramifications. One area that may be substantially affected by the consequences of nasal obstruction in early development is neurological development. Given this concern, a recent study showed that nasal obstruction during the early developmental years can cause changes in the brain. Research based on animal models demonstrated that when chronic nasal obstruction occurs, cerebellar function is impaired, and with motor deficits, there will also be depressive-like behaviors. These physiological and functional outcomes appear to be associated with altered synaptic pruning and cerebellar neuron competencies [1].

Beyond its neurological aspects, NOS is also strongly linked to obstructive sleep apnea (OSA), a disorder of recurrent upper airway obstruction during sleep. This is significant because OSA carries risks to cardiovascular, metabolic, and neurocognitive health, and effective treatment of nasal obstruction may alleviate the burden of sleep-related morbidity [2, 3].

A relationship has also been demonstrated between NOS and chronic rhinosinusitis (CRS). Patients with both CRS and OSA have a higher comorbidity burden of disease and need more intensive treatments than those without this comorbidity in many cases. This may be endoscopic sinus surgery or prolonged use of antibiotics and steroids to treat both conditions. Therefore, effective treatment of nasal obstruction may alleviate the treatment and clinical burden of these two comorbid conditions [4].

From an epidemiological viewpoint, NOS is very common across all ages and is often comorbid with other diseases associated with obstructive symptoms (e.g., asthma, obesity, gastroesophageal reflux disease). These comorbidities might increase obstructive symptoms and their management; hence, it is important to consider any therapeutic approach in a holistic way, which includes consideration of all the clinical elements [5,6].

NOS does have a significant impact on the quality of life. Living with nasal obstruction day-to-day results in poor sleep quality, excessive daytime sleepiness, and impairment associated with cognitive function. These are of particular concern in patients with OSA, as nasal obstruction might inhibit the effectiveness of treatments like continuous positive airway pressure therapy (CPAP) [7, 8].

For the pediatric population, NOS has a different meaning because it may serve as a precursor to childhood OSA. NOS is frequently related to hypertrophy of lymphoid tissues in the upper airways. This demonstrated relationship reinforces the importance of timely diagnosis and treatment of NOS before it leads to significant deteriorating consequences for respiratory and developmental health [6].

The objective of this study is to comprehensively examine the existing scientific information about NOS, including its clinical aspects, common causes, diagnostic methods, and treatments, in order to encourage an informed, comprehensive, personalized approach to its diagnosis and management.

## Review

Methods

This review was formed using a specific but flexible search strategy to gain a comprehensive account of knowledge regarding NOS and its linked clinical approaches. Three databases with broad biomedical coverage in the world were used for this purpose, namely PubMed, Scopus, and ScienceDirect. The search was limited by date to the five years from 2020 to 2025 and could be custom-tailored to English and/or Spanish. The descriptors of terms included practically all correct words, such as nasal obstruction, nasal airflow, OSA, CRS, pediatric nasal obstruction, and nasal surgery, quickly revamped with the Boolean operator adjustments so that more precise clinical, pathophysiological, and therapeutic topics could emerge from the results related to NOS.

Constraining systematic analysis with rigid rules and strict definitional criteria may result in a limited body of literature and the omission of clinically useful, valid, reputable, and recent evidence. Therefore, a selective and adaptive criterion was applied, emphasizing potential clinical utility, recentness of findings, and methodological rigor. Included sources comprised original research articles, systematic reviews, evidence-based clinical practice guidelines, and expert consensus statements published by recognized scientific societies. Studies were considered eligible if they addressed nasal obstruction or related conditions in pediatric or adult populations, with a focus on diagnosis, pathophysiology, or treatment strategies, whether medical or surgical.

Exclusion criteria included duplicate publications, studies with limited or no clinical relevance, and those that lacked methodological quality, such as opinion pieces, isolated case reports, editorials, or abstracts without full texts. Animal studies were excluded unless they offered clear translational value relevant to human clinical practice.

**Figure 1:**
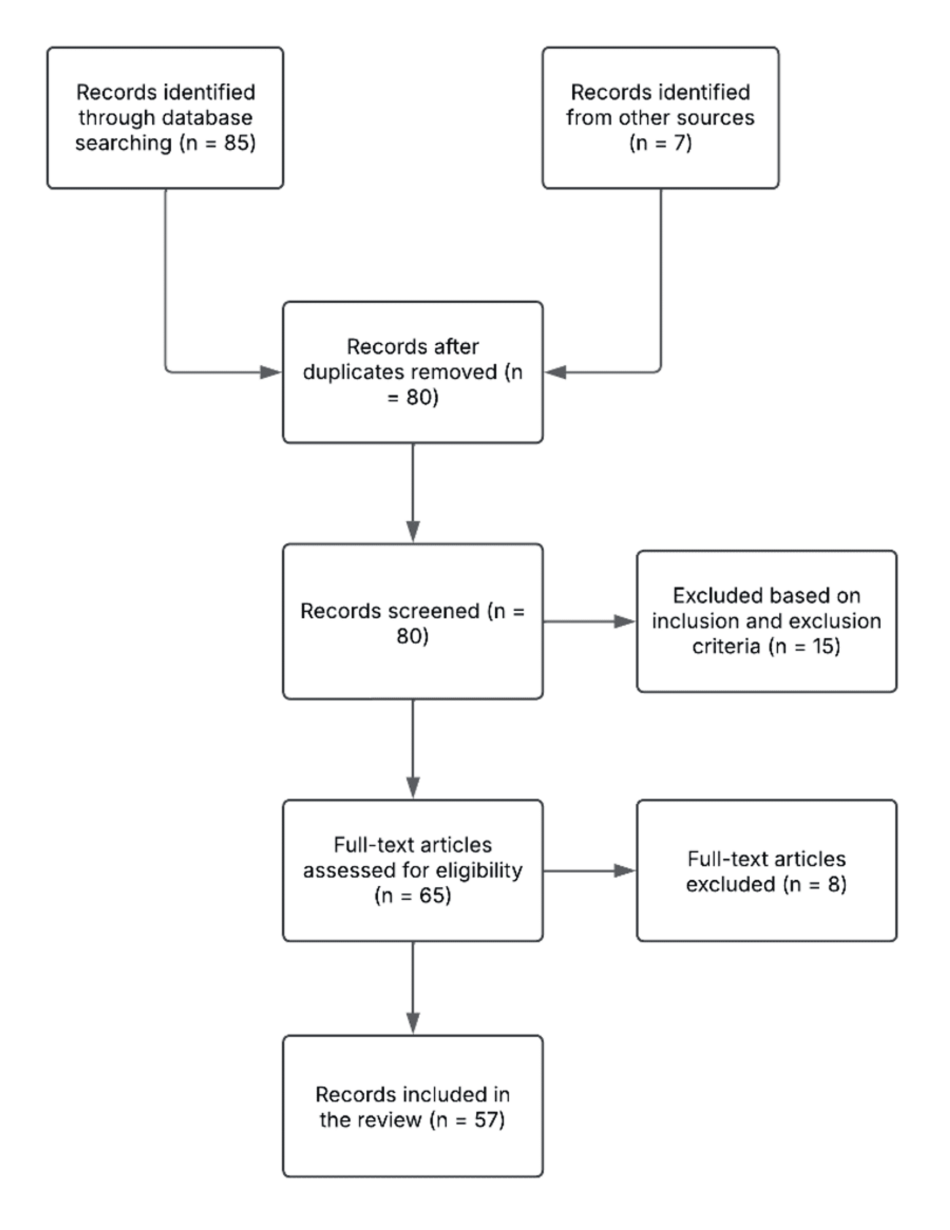
A flow diagram of the study selection process

The resulting data set included a large number of observational studies, explanatory narrative reviews, experimental studies using animals, and some evidence-based clinical practice guidelines (Figure 1). This variety afforded a generalized and critical angle on NOS, or its impacts on pathophysiology and clinical-therapeutic strategies used for complete management.

It should be noted that artificial intelligence (AI) assistance was used as an auxiliary tool when constructing this manuscript. The AI was used to help create a reliable structure, synthesized and edited for clarity, without supplanting the critical thinking and educational judgment of the authors.

Nasal anatomy and physiology

The nasal breathing process relies on a complex anatomical system that operates in a synergistic way to create air movement, thermal conditioning, and a sense of smell. The nasal cavity is divided by the septum: the nasal cavity breaches and opens to the nasopharynx side and contains the turbinates, which are mucosa-covered bony structures within the nasal cavity. The turbinates manage air flow and act to humidify and warm air flow [9, 10].

**Figure 2:**
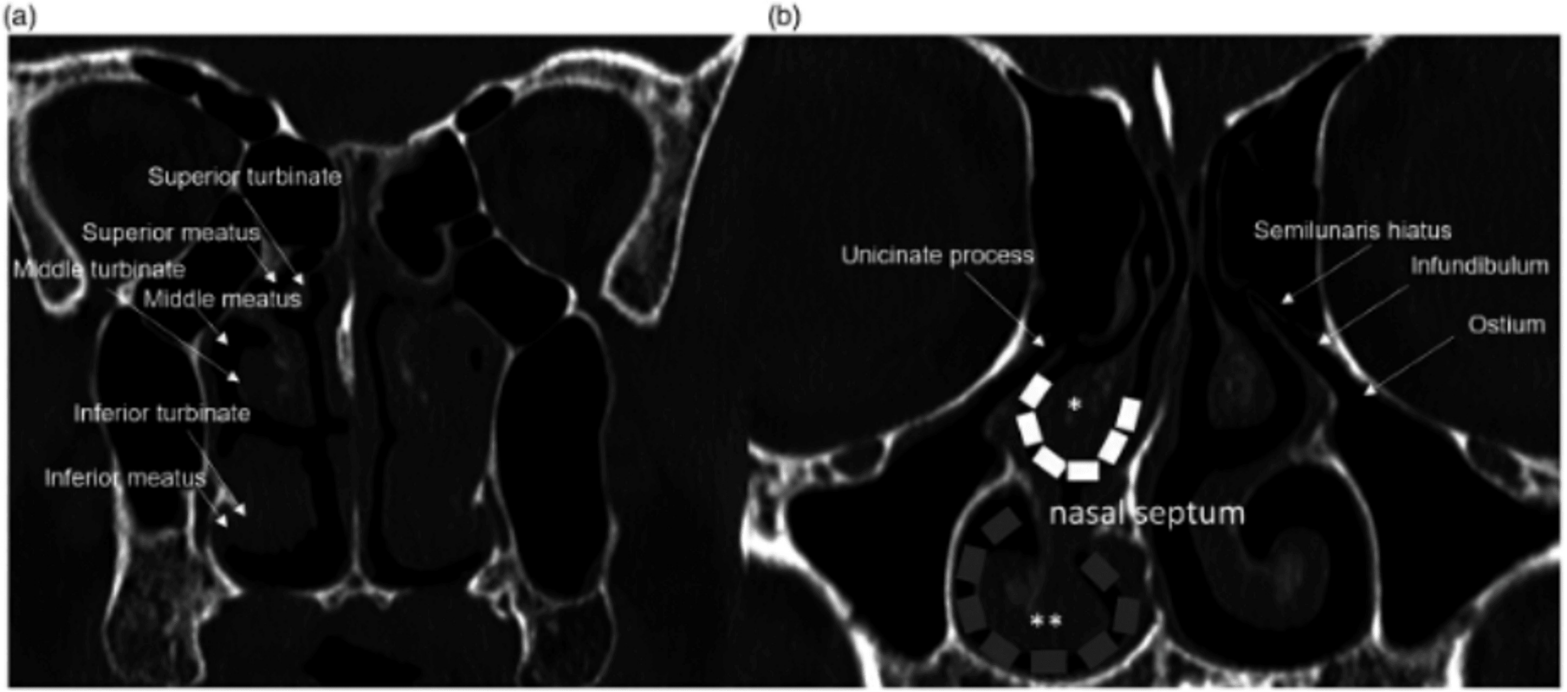
Coronal CT views of the nasal cavity anatomy and turbinate-related obstruction (a) Right nasal cavity showing normal anatomical structures: inferior, middle, and superior turbinates and their respective meatuses. The left nasal cavity is obstructed due to nasal polyposis. (b) Key anatomical landmarks for sinus ventilation and drainage, including the uncinate process, semilunar hiatus, infundibulum, and ostium. A white dashed line indicates the middle meatus; a grey dashed line outlines the inferior meatus; a single asterisk denotes the middle turbinate; and a double asterisk indicates the inferior turbinate. Figure reproduced from Cellina M, Gibelli D, Cappella A, Martinenghi C, Belloni E, Oliva G. *Nasal cavities and the nasal septum: Anatomical variants and assessment of features with computed tomography.* The Neuroradiology Journal (Volume 33, Issue number 4). pp. 340-7. Copyright © 2020, Sage Publications. Reproduced with permission of Sage Publications [11].

Figure 2 illustrates the detailed anatomical relationships within the nasal cavity, including the inferior, middle, and superior turbinates, as well as their respective meatuses. These structures play a fundamental role in airflow regulation, humidification, and mucociliary clearance. As shown in the left nasal cavity (Figure 2a), nasal polyposis can significantly obstruct airflow and compromise sinus ventilation. In contrast, the right nasal cavity shows a normal anatomical configuration. Figure 2b highlights critical anatomical landmarks such as the uncinate process and semilunar hiatus, which are frequently involved in the pathophysiology of chronic rhinosinusitis and nasal obstruction [11].

The nasal valves (internal and external) are also the dynamic pressures that air needs to encounter. The nasal valves act as resistances that control and regulate pressure and the direction of the airflow. Their collapse or narrowing can lead to a decrease in airflow, even with no apparent anatomical alterations [9, 10]. The olfactory cleft, a part of the most superior aspect of the nasal cavity, serves to detect odors via a pattern of airflow, so any anatomical or functional change can hinder odor perception [12]. The nose has important functions that include filtration, humidification, and olfaction. Filtration by the intricately designed internal structures of the air passages, filtering particles and pathogens [13]. Humidification and warming of the air depend on the abundant vascularity of the mucosa, thus protecting the lower respiratory tract [13, 14]. An intact anatomy of the olfactory cleft and sufficient airflow are necessary for olfactory function. A disruption in either will decrease the activation of olfactory receptors and sense of smell [15].

From a pathophysiological perspective, nasal obstruction may occur because of anatomical considerations such as septal deviation, turbinate hypertrophy, or valvular collapse [9, 10] or inflammation such as CRS or allergic rhinitis, in which mucosal edema and epithelial dysfunction maintain the obstruction [15, 16]. In some instances, the sensation of nasal obstruction may not be due to any visible structural changes but rather due to dysfunction of the trigeminal nerve (which perceives airflow). There is no question that this sensory dysfunction represents a particular diagnostic quandary [17].

Etiology of NOS

The pathophysiology of NOS is caused by both structural and functional causes, which will often be present together, and with potential external causal factors. Structural causes include things such as nasal septal deviation with resulting mechanical obstruction of airflow. Patients who undergo a nasoseptal intervention, either a septoplasty with or without turbinectomy or a septectomy and turbinectomy, have improved nasal patency as well as quality of life after nasoseptal surgery, pending their type of deviation and technique [18,19]. It is very common for inferior turbinate hypertrophy to accompany septal deviation, further increasing nasal resistance. Radiofrequency ablation has been shown to lessen the size of hyperplastic turbinates and relieve symptoms, although there may be a higher incidence of complications when ablating turbinates if the patient has a nasal septal deviation [18, 20].

Nasal polyps, which appear more commonly among those with CRS, also obstruct the nasal passages. They are often derived from the T2 endotype, which is characterized by eosinophilic inflammation. For patients with T2 endotype allergic eosinophilic rhinosinusitis, monoclonal antibodies targeting type 2 inflammation have been shown to decrease polyp volume and improve symptoms of underlying sinusitis [21-23]. Allergic rhinitis is among the most common functional causes and is induced by environmental allergens. As a result, for patients with a septal deviation, the diagnosis can become more cumbersome due to the presence of asymptomatic sensitization [24]. Non-allergic rhinitis occurs in response to non-immunologic events (irritants, weather, hormonal) [1].

Dysfunction of the nasal valves, either internal or external, can also cause significant obstruction, even without overt structural changes. Functional surgery for nasal valve defects (to correct them) has restored airflow [9]. Lastly, obstructions also worsen from smoking and environmental pollution, causing chronic inflammation and mucosal edema, and less effective air filtration and humidification [25]. Respiratory infections, whether acute or chronic, as the latter remain a risk factor for chronic rhinosinusitis [21].

Clinical manifestations and quality of life

NOS manifests and progresses with either respiratory or sensory disorders and has a significant impact on quality of life. The prolonged nasal congestion and/or rhinorrhea seen in NOS cases requires mouth breathing to compensate, which has been documented to impair taste and sense of smell [26]. Consequently, hyposmia disables not only sensory enjoyment but also interferes with daily living, contributing to an unfavorable perception of their health status [27]. Mouth breathing, as a compensatory mechanism, has many of its own ramifications. Along with all the symptoms associated with nasal obstruction, mouth breathing creates additional symptoms, including dry mouth, pharyngeal discomfort, and a higher likelihood of recurrent respiratory infections. In children, mouth breathing can affect orofacial development quality and lead to fatigue in adults [28]. Sleep is by far the most affected area. Nasal obstruction is associated with sleep disorders such as snoring and OSA and impairs the quality of sleep and oxygenation, leading to daytime sleepiness and potential cardiovascular and neurocognitive complications [3, 29]. Interventions to treat nasal obstruction, such as radiofrequency ablation, have improved sleep quality and reduced snoring [26].

In addition to the symptom of difficulty breathing, NOS can have a major role in quality of life, as it may limit vitality, focus, and mood. Disturbances in sleep that can be from NOS may lead to impaired executive function, including memory and attention, and can be particularly deleterious in children, where they may influence development socially and academically [13].

Functionally, persistent nasal obstruction can include symptoms such as nasal congestion and decreased smell sensitivity (hyposmia) and can lead to poor sleep quality, which may also represent productivity loss and increased fatigue, which can negatively affect your ability to perform daily activities and to have life satisfaction overall [30]. In children, even mild nasal obstruction has also been linked to behavior problems, poor academic performance, and low quality of life; as such, it is concluded that early diagnosis and treatment are essential [13]. If NOS negatively impacts sleep, and therefore quality of life, management should be tailored to include considerations for the severity of OSA if it exists, comorbidities, and individual needs. Further, often a combination of treatments that may specifically include medical treatment and different lifestyle changes, like CPAP therapy, may be required [30,31].

Diagnostic evaluation

**Figure 3:**
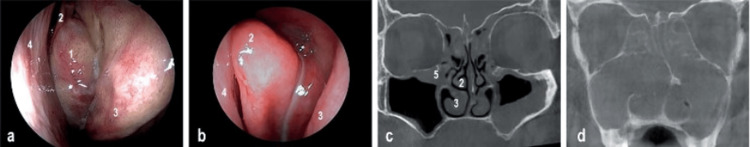
Diagnostic evaluation of chronic rhinosinusitis (a) Endoscopic image of CRSwNP showing nasal polyps and mucus secretion; (b) Endoscopic view of CRSsNP with mucosal edema; (c) CT scan with osteomeatal complex obstruction in CRSsNP; (d) CT scan showing diffuse polyposis in CRSwNP (1 = nasal polyps, 2 = middle turbinate, 3 = inferior turbinate, 4 = nasal septum, 5 = ostiomeatal complex). CRSwNP: chronic rhinosinusitis without nasal polyps Figure reproduced from Hildenbrand T, Milger-Kneidinger K, Baumann I, Weber R. *The Diagnosis and Treatment of Chronic Rhinosinusitis. *Deutsches Ärzteblatt International (Volume 121, Issue number 19)*.* pp 643-653. Copyright © 2008, Deutscher Ärzteverlag GmbH. Reproduced with permission of Deutscher Ärzteverlag GmbH [34].

Diagnosing NOS involves a thorough clinical assessment in which medical history and physical examination have essential roles in detecting the causes of nasal obstruction, including septal deviation, CRS, or nasal polyps [32, 33]. Physical findings are often poorly correlated with the severity of obstruction as reported by the patient, thus indicating a need for further diagnostic measures (Figure 3) [32].

Symptom severity scales have good recognition among different diagnostic measures; among them, a series of score scales have particularly depicted their purpose. The Nasal Obstruction Symptom Evaluation (NOSE) scale, for example, has been well validated to assess the severity of symptom reporting and progression after some treatments, such as septoplasty [35], which has been validated on multiple normative values from various populations [36, 37]. The Sino-Nasal Outcome Test-22 (SNOT-22) questionnaire and its 22-item version have been utilized primarily in chronic rhinosinusitis, providing assessment of quality of life as impacted by symptoms from CRSs, and it relates well with the combined score of the Visual Analog Scale (VAS), indicating its relevance in clinical assessment [38, 39]. The VAS is a useful scale to quantify some symptoms, including nasal congestion, and the relationship with SNOT-22 is strong, indicating its clinical relevance for symptom tracking in chronic patients [39].

**Figure 4:**
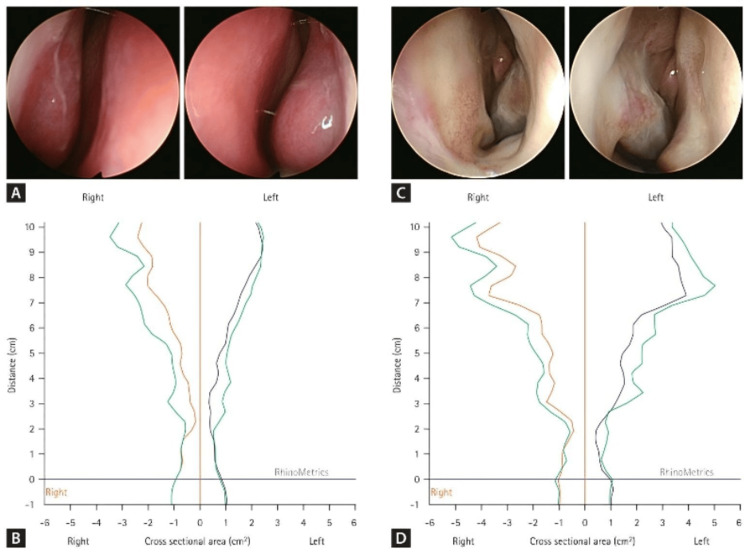
Endoscopic and acoustic rhinometry findings in patients with anterior nasal airway narrowing. Figure reproduced from: Min HJ, Park JY. *Usefulness of nasal cavity evaluation before high-resolution esophageal manometry in high-risk patients*. The Korean Journal of Internal Medicine. 2024 Jan;39(1):86-94. Licensed under CC BY 4.0. [40].

Nasal endoscopy is among the adjunctive options, since the procedure allows for direct assessment of anatomic or inflammatory lesions like polyps (Figure 4) [36]. Rhinomanometry and acoustic rhinometry provide a more objective assessment of nasal airflow and resistance, but their ability to correlate with a subjective symptom is limited, so we recommend their use in conjunction with subjective scales [33]. Last, CT can assist with the assessment of the anatomy of the paranasal sinuses of complicated cases or surgical patients, but is not recommended routinely without a specific clinical indication [33].

**Figure 5:**
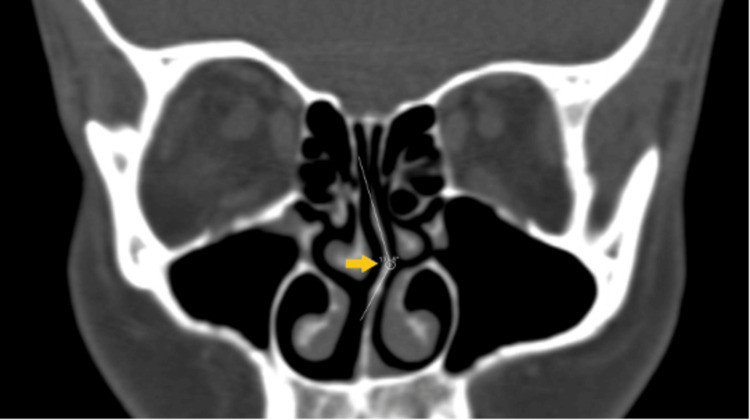
Coronal computed tomography scan showing the measurement of the septal deviation curve angle A relevant anatomical alteration in patients with nasal obstruction was noted. Figure reproduced from Bekin Sarikaya P, Bayar Muluk N. *Relationship between nasal septal deviation angles and turbinates: a computed tomography study*. Cureus. 2023;15(2):e35253. Licensed under CC BY 4.0 [41].

Figure 5 illustrates a coronal CT scan demonstrating the measurement of the septal deviation curve angle, a key anatomical alteration in patients with nasal obstruction. Tomographic evaluation allows for precise assessment of structural deviations that may not be fully appreciated on physical examination.

Current management strategies

Historically and presently, decongestants and antihistamines are common medications for allergic rhinitis, a leading cause of nasal obstruction, among other medical and surgical treatments. Antihistamines work at the level of the H1 receptor and reduce signs and symptoms, rhinorrhea, sneezing, nasal pruritus, and more. Decongestants work by reducing nasal mucosal inflammatory effects to ease airflow [42, 43]. 

Intranasal corticosteroids are another option to consider for patients with CRS with nasal polyposis. Intranasal corticosteroids are also applicable for allergic rhinitis and have their own unique anti-inflammatory effect with a dramatic reduction in nasal obstruction. Intranasal corticosteroids can be delivered via standard nasal spray and other methods like intranasal stents, demonstrating clinically relevant effects on obstruction [42,44].

Allergen-specific immunotherapy serves as a causal treatment option for allergic rhinitis. This is coupled with surgery, like inferior turbinate reduction, that provides significantly superior symptomatic relief compared to immunotherapy alone, particularly in patients with sustained allergic sensitization [42]. Nasal saline irrigation, as a complementary modality, is an effective non-pharmacological treatment. This method helps to cleanse the nasal passages, decrease inflammatory load, and relieve congestion, making it a great adjunct to daily maintenance therapy [42]. When medical therapies are ineffective or anatomically inappropriate, surgical treatment is warranted. Septoplasty is the standard surgical procedure to amend nasal septal deviation influencing airflow and decrease nasal resistance, with favorable results in allergic rhinitis patients [45].

Septoplasty is often used in conjunction with tuboplasty or inferior turbinate reduction. Tuboplasty and inferior turbinate reduction are both indicated in patients with turbinate hypertrophy. The combination of septoplasty and tuboplasty has been shown to effectively address nasal obstruction and improve patient satisfaction [46, 47]. Functional endoscopic sinus surgery (FESS) is also a minimally invasive option for patients suffering from chronic rhinosinusitis or nasal polyps. This surgical option helps improve sinus drainage, and its pathophysiological benefits reduce upper airway obstruction, with clinically significant results [46].

Reconstruction of the nasal valve is available as another surgical option. This option is indicated when there are structural abnormalities that reduce the functionality of the valvular area and/or when other medical treatments have been unsuccessful. The surgical procedure aims to restore the functional support of both internal and external nasal valves [48]. 

**Figure 6:**
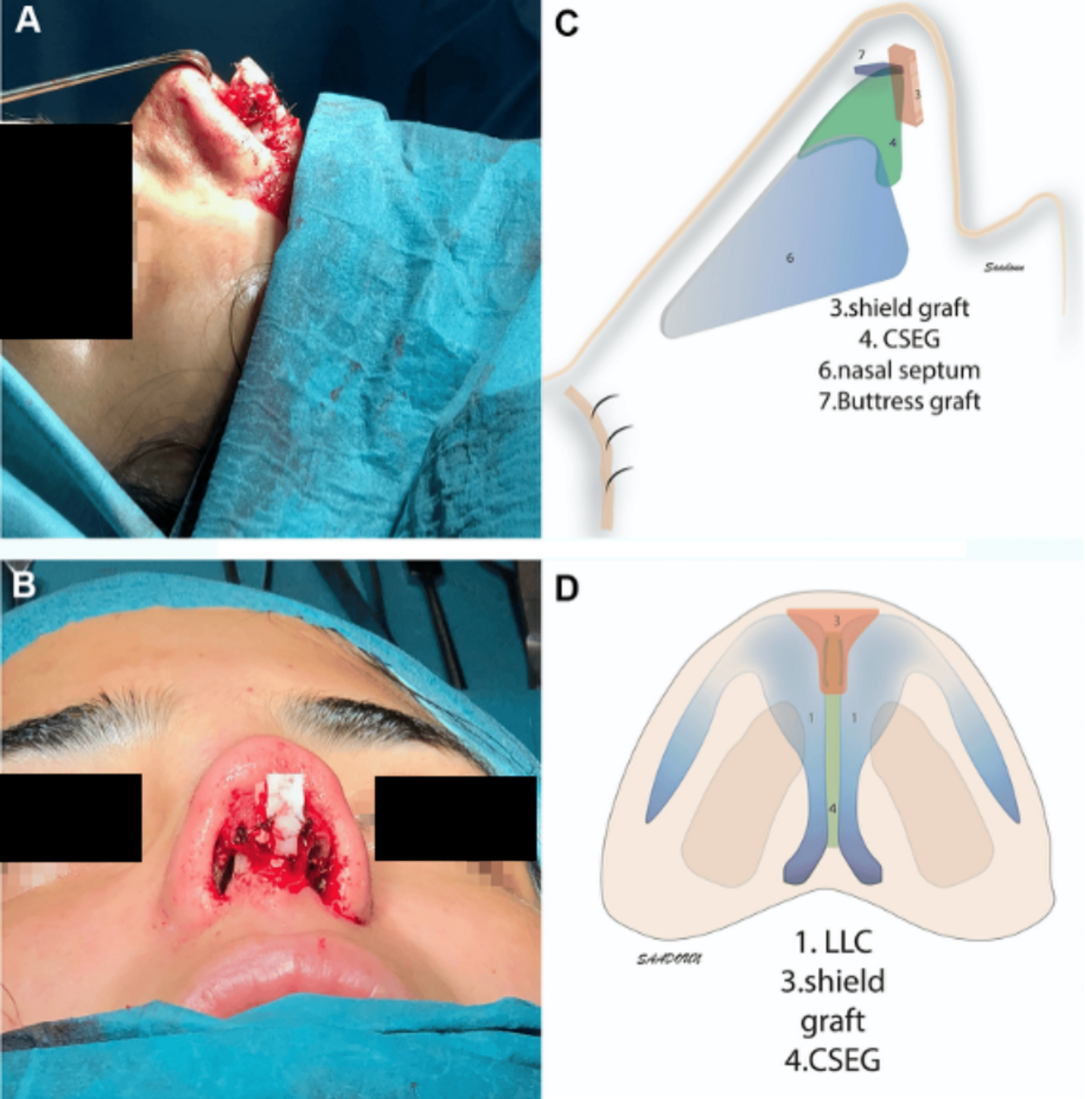
Intraoperative and schematic views of nasal valve reconstruction using shield grafts (SiG), caudal septal extension grafts (CSEG), and buttress grafts. (A–B) Intraoperative views; (C–D) Schematic depictions from lateral and basal perspectives. Figure reproduced from: Saadoun R, Veit JA.* Revision septorhinoplasty: An illustrative case report*. Ear, Nose & Throat Journal. 2020;100(10_suppl):924S–929S. https://doi.org/10.1177/0145561320925964. Licensed under CC BY 4.0 [49].

Among the surgical options for nasal valve reconstruction, the use of structural grafts such as shield grafts (SiG), caudal septal extension grafts (CSEG), and buttress grafts has demonstrated effectiveness in restoring support and improving nasal airflow. Figure 6 illustrates both intraoperative and schematic views of these grafts, providing visual guidance on their anatomical placement and configuration.

As NOS is multifactorial, a multidisciplinary approach is essential. Coordination of care between otolaryngology, allergology, and pulmonology specialists is essential to provide adequate treatment to the respective etiological components of the disease. Therefore, when relevant, if the patient also has associated comorbidities, adequate management of those diseases (e.g., asthma, obstructive sleep apnea/hypopnea syndrome) also must occur to address the totality of symptoms [42].

Special considerations and vulnerable populations

Adenoid hypertrophy is an important etiological aspect of OSA in pediatrics and nasal obstruction. Adenoidectomy improves airflow and objective clinical symptoms, since postoperative airflow showed an improvement in olfaction [50, 51]. Patients with this condition also frequently demonstrate nasal dysbiosis during a nasal examination, and dysbiosis may mediate both inflammatory responses and adenotonsillar hypertrophy, which contribute to allergic rhinitis [52]. Although adenotonsillectomy is a frequent treatment option in children, patients who have obesity or neuromuscular conditions are still at risk for persistent OSA, and CPAP therapy can be a valid alternative treatment option [53].

CRS is a common condition often found in geriatric populations, with the possibility of associated bacterial infections. The unique nasal microbiota of these individuals also warrants the use of individualized treatment plans [54]. While there are challenges to mobile health technologies that need to be considered in the geriatric population, some studies have shown possibilities for benefits when they are used in the clinic [55].

Patients with chronic respiratory conditions such as asthma or chronic obstructive pulmonary disease (COPD) may have symptoms like those who have nasal pathologies. Treating these patients holistically means treating the upper airway as well as the lower respiratory tract [56]. In this group of patients, procedures such as functional endoscopic sinus surgery are commonly undertaken, and while the procedures may not be the best option in cases of multimorbidity, they may need to be repeated [57].

## Conclusions

NOS is a complicated issue that arises from multiple origins and has a significant clinical, physiological, and functional impact. An effective diagnostic strategy must utilize both clinical evaluation and subjective and objective methods, as physical findings do not always correlate with the severity perceived by the patient. An integral component of effective treatment involves a thorough understanding of the structural, functional, and inflammatory causes and tailoring personalized treatment plans that utilize combination medications (e.g., intranasal corticosteroids and antihistamines, immunotherapy, etc.) followed by surgical interventions if conservative management fails to prove efficacious. Septoplasty, tuboplasty, and endoscopic sinus surgery offer positive clinical outcomes, especially when integrated into a multidisciplinary approach.

Populations vulnerable to impactful conservative management of NOS include pediatric, aged, and patients with chronic respiratory disease (e.g., asthma or COPD). These populations will require personalized treatment approaches for various reasons. The pediatric population has issues such as adenoid hypertrophy and nasal dysbiosis. The aged population has varying nasal microbiota and technology involvement challenges. Patients with pulmonary comorbidities require a holistic approach that engages the upper airways and lower airways either via education and/or additional referral.
